# Batesian mimicry has evolved with deleterious effects of the pleiotropic gene *doublesex*

**DOI:** 10.1038/s41598-020-78055-1

**Published:** 2020-12-07

**Authors:** Shinya Komata, Tasuku Kitamura, Haruhiko Fujiwara

**Affiliations:** Department of Integrated Biosciences, Graduate School of Frontier Sciences, The University of Tokyo, Kashiwa, Chiba 277-8562 Japan

**Keywords:** Batesian mimicry, Evolutionary biology

## Abstract

Dimorphic female-limited Batesian mimicry in the swallowtail butterfly *Papilio polytes* is regulated by the supergene locus *H*, harbouring the mimetic (*H*) and non-mimetic (*h*) *doublesex* (*dsx*) gene. In the present study, we demonstrated that *dsx-H* negatively affects the number of eggs laid, hatching rate, larval survival rate, and adult lifespan. When crossed with *hh* males, the number of eggs laid of mimetic females (genotype *HH*) was lower than that of non-mimetic females (*hh*). Moreover, *hh* and *Hh* females laid fewer eggs when crossed with *HH* males. The hatching and larval survival rates were lower when both female and male parents harboured *dsx-H*. The adult lifespan of *HH* females was shorter than that of *hh* females, while it was similar in males regardless of the genotype. These findings suggest the presence of a cost–benefit balance of Batesian mimicry, which is evolved to avoid predation but is accompanied by physiological deficits, in this species.

## Introduction

**Figure 1:**
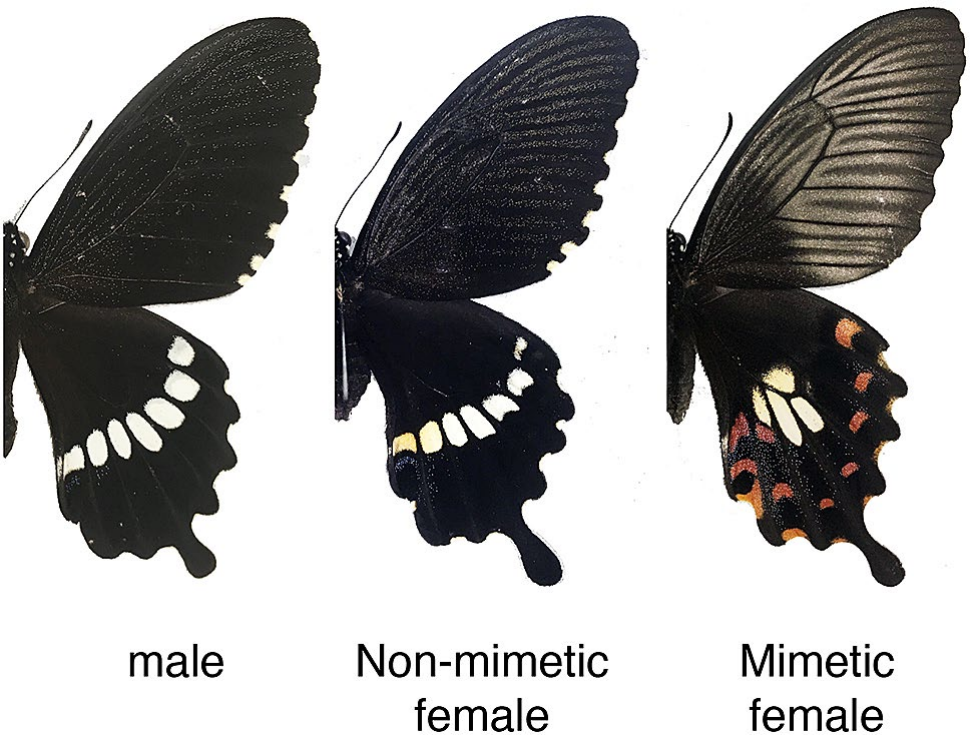
Wing patterns of adult male and non-mimetic and mimetic females of *Papilio polytes*. Photos by Shinya Komata. This figure was generated using Adobe Photoshop 2020 and Illustrator 2020 (Adobe Systems Incorporated, San Jose, CA, USA).

In Batesian mimicry, palatable mimics avoid predation by resembling unpalatable models, providing a compelling evidence of evolution by natural selection^1^. One of the most intriguing types of Batesian mimicry in butterflies is the polymorphic female-limited mimicry, in which females show both mimetic and non-mimetic forms, while males are uniformly non-mimetic^2^ (Fig. 1). Recently, the genetic basis of female-limited Batesian mimicry was revealed in *Papilio polytes*, *Papilio memnon*, and *Papilio dardanus*^3–6^. In *Papilio polytes*, a single autosomal region containing the sex-determinant gene *doublesex* (*dsx*), a ubiquitously expressed transcript (*UXT*), and a long noncoding RNA gene (*U3X*) constitutes a supergene locus that switches mimetic and non-mimetic forms^4^. This supergene locus with mimetic (*H*) and non-mimetic (*h*) alleles is likely protected against recombination by a chromosomal inversion (130 kb)^4^. In this species, mimetic females resemble the unpalatable model *Pachliopta aristolochiae*, and the mimetic allele (*H*) is dominant to the non-mimetic one (*h*)^7^ (Fig. 1). Functional analyses using electroporation-mediated RNA interference (RNAi) revealed that the mimetic-type *dsx* (*dsx-H*) is a key factor driving the mimetic phenotype whilst simultaneously repressing the non-mimetic phenotype in *Papilio polytes*^4,8^.

Although the evolution of Batesian mimicry has endowed the selective advantage of reduced predation, it may also be associated with physiological trade-offs, which may affect the establishment and maintenance of female-limited polymorphism^9,10^. The evolution and maintenance of colour polymorphism to avoid predation have been studied in diverse contexts, such as natural selection, sexual selection, and life-history trade-offs^11–14^. In female-limited polymorphic mimicry, negative frequency-dependent selection (NFDS), through which the advantage of mimetic forms decreases as their frequency increases, is indispensable to maintain polymorphism^10^. The advantage of Batesian mimicry decreases when the relative abundance of mimics to that of unpalatable models increases, because predators more readily learn that Batesian mimics are palatable when they are more common^15,16^. The relative frequency of mimetic to non-mimetic forms within a species is at an equilibrium level at which both forms have equal fitness via NFDS^17^. This equilibrium level may in turn be influenced by the potential effects of mimicry, such as male mate choice and physiological deficits^9,17^. Moreover, this level shifts to a point at which the frequency of mimetic forms is lower when there are some costs of mimetic form than when only NFDS determines the fitness. In *Papilio polytes*, the importance of NFDS has been repeatedly reported^9,17,18^. On five islands in Okinawa, Japan, the abundance of mimics of *Papilio polytes* was strongly correlated with the abundance of model, which is expected under NFDS^18^. Regarding male mate choice in *Papilio polytes*, male preference for non-mimetic females is pivotal for maintaining polymorphic mimicry^19^. A hypothesis regarding physiological costs has been examined in a greenhouse experiment with *Papilio polytes*: non-mimetic females live longer than mimetic females and the production of a mimetic form is physiologically costly^9^. In addition, the survival rate in juvenile period decreased over generations (F0–F2) in lines derived from mothers with mimetic phenotypes, suggesting a mild deleterious effect of *dsx-H*^20^. In these experiments, however, the associations of the mimetic allele (*dsx-H*) with various physiological characteristics, including survival rate and adult longevity, were not explored, and the deleterious effects of *dsx-H* on these traits remain unclear. *dsx-H* in the supergene likely accumulates deleterious recessive mutations due to limited recombination^21^. In this case, homozygous-dominant (*HH*) individuals would be lethal or show reduced fitness (e.g., genetic load). The deleterious effects of inversions have been reported in many studies, which showed that the homozygous form is lethal^21–24^.

Because the pleiotropic gene *dsx* regulates sex differentiation at various developmental stages in many holometabolous insects^25–27^, specific mutations or improper expression of mimetic *dsx-H* may produce deleterious effects on traits other than mimetic ones. Male- and female-specific isoforms of *dsx* regulate several genes involved in male and female reproductive functions, such as genitalia development, yolk protein production, and fat body deposition in females^27–33^. In *Drosophila melanogaster*, Shen et al.^34^ reported that sex-determinant genes, including *dsx*, can affect adult lifespan even after the completion of morphological changes associated with sex differentiation. In *Papilio polytes*, there are three female-specific isoforms (F1–F3) of *dsx-H,* while there is only one male-specific isoform, although the expression of *H* and *h* alleles of *dsx* is very low in the wings of *Hh* males^4^, Kitamura, unpublished data.

To examine whether the evolution of Batesian mimicry in *Papilio polytes* is accompanied by physiological deficits, we explored the effects of *dsx*-*H* on various traits other than mimetic ones using three experiments each (1) exploring the frequency of *dsx* genotypes in the wild, (2) examining the effects of parental genotypes on fecundity and larval performance, and (3) examining the effects of genotypes on larval development and adult lifespan. The experimental scheme is shown in Supplementary Fig. S1. In the present study, we demonstrated that the homozygous-dominant (*HH*) genotype is not lethal in the wild, but *dsx-H* negatively affects fecundity, larval performance, and adult lifespan. Furthermore, potential mechanisms underlying these deleterious effects of *dsx-H* on physiological traits are discussed.

## Results and discussion

First, we examined the *dsx* genotypic frequency of individuals collected from the wild. We genotyped the *dsx* locus of 104 mimetic females and 118 males collected from Ishigaki Island, Okinawa, Japan. The genotypic frequencies of mimetic females (*HH*:*Hh*) and males (*HH*:*Hh*:*hh*) were 6:98 and 4:36:78, respectively. The genotypic frequency of males did not significantly deviate from the expected frequency according to the Hardy–Weinberg equilibrium (Chi-square test; *P* = 0.951). The homozygous-dominant (*HH*) genotype was not lethal in males and females. The percentage of *HH* individuals in mimetic females and non-mimetic males were 5.8% and 10.0%, respectively, but this percentage did not significantly differ between females and males (Fisher exact test; *P* = 0.46).

**Figure 2:**
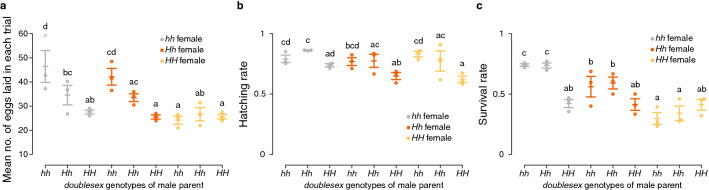
Mean number of eggs laid in each trial (**a**), hatching rate (**b**), and larval survival rate (**c**) in each *doublesex* genotype of parents. We used crosses between all combinations of *dsx* genotypes. Parental females were captured in the wild, and the offspring were used for crossing. Grey plots, *hh* female parents; orange plots, *Hh* female parents; yellow plots, *HH* female parents. Middle bar represents mean, and upper and lower bars indicate standard error. Different letters indicate significant differences between groups (Tukey post hoc test, P < 0.05; Supplementary Table S5). (**a**) Y-axis shows the mean number of eggs laid in each egg correction trial, and plots show the mean number of eggs laid by each female.

Next, we compared the number of eggs laid, hatching rate, and larval survival rate using crosses between females and males with all combinations of *dsx* genotypes (*hh*♂ × *hh*♀, *Hh*♂ × *hh*♀, *HH*♂ × *hh*♀, *hh*♂ × *Hh*♀, *Hh*♂ × *Hh*♀, *HH*♂ × *Hh*♀, *hh*♂ × *HH*♀, *Hh*♂ × *HH*♀, and *HH*♂ × *HH*♀). We obtained a total of 2,286 eggs and calculated the mean number of eggs laid. There were significant differences amongst the genotypes of female and male parents and their interactions (Fig. 2a, Supplementary Tables S1, S2). *HH* females laid significantly fewer eggs than *hh* females when crossed with *hh* or *Hh* males as well as than *Hh* females when crossed with *hh* males (Fig. 2a; Tukey post hoc test, P < 0.05; Supplementary Table S5). *hh* females crossed with *hh* males laid more eggs than those crossed with *HH* and *Hh* males (Tukey post hoc test, P < 0.001; Supplementary Table S5), while *Hh* females crossed with *hh* males laid more eggs than those crossed with *HH* males (Tukey post hoc test, P < 0.001; Supplementary Table S5). Although the exact reason for this result remains unknown, male parents carrying the *H* allele may have reduced fecundity through nutritional investment in spermatophore, which affects the reproductive output of females^35^. A total of 1767 eggs hatched, and the overall hatching rate was 77.3%. Variations in hatching rate could also be explained by the genotypes of female and male parents and their interactions (Fig. 2b, Supplementary Tables S1, S2). The hatching rate of eggs laid by *hh* females crossed with *Hh* males was higher than that of eggs laid by *hh*, *Hh*, and *HH* females crossed with *HH* males (Fig. 2b; Tukey post hoc test, P < 0.01; Table S5). Meanwhile, the hatching rate of eggs laid by *hh* females crossed with *hh* males was higher than that of eggs laid by *Hh* and *HH* females crossed with *HH* males (Tukey post hoc test, P < 0.05; Table S5). The hatching rate of eggs laid by *HH* females crossed with *HH* males was significantly lower than that of eggs laid by *hh*, *Hh*, and *HH* females crossed with *hh* males (Tukey post hoc test, P < 0.01; Table S5). Finally, we obtained 1,017 pupae and calculated larval survival rate. The overall survival rate was 57.6%. Variations in larval survival rates could be explained by the genotypes of female and male parents and their interactions (Fig. 2c, Supplementary Tables S1, S2). Survival rates of larvae of *hh* females crossed with *hh* and *Hh* males were significantly higher than those of larvae of other females (Fig. 2c; Tukey post hoc test, P < 0.05; Table S5). Meanwhile, survival rates of larvae of *HH* females crossed with *hh* and *Hh* males were significantly lower than those of larvae of *hh* and *Hh* females crossed with *hh* and *Hh* males (Fig. 2c; Tukey post hoc test, P < 0.05; Table S5). Although we explored the effects of parental genotypes in this study, offspring genotypes may be more important for larval survival rate than parental genotypes. However, our experiments could not distinguish between the effects of parental and offspring genotypes. For instance, larvae of *hh* females crossed with *hh* males showed significantly higher survival rate than larvae of *HH* females crossed with *HH* males (Tukey post hoc test, P < 0.001; Table S5). This difference can be explained by both parental and offspring genotypes. Meanwhile, when we compared the larvae of *hh* females crossed with *Hh* males to those of *Hh* females crossed with *hh* males, the segregation of *dsx* genotypes was identical (*hh*:*Hh* = 1:1) but the survival rate was significantly different (Tukey post hoc test, P < 0.001; Supplementary Table S5), suggesting a pivotal role of parental genotypes in offspring survival.

**Figure 3:**
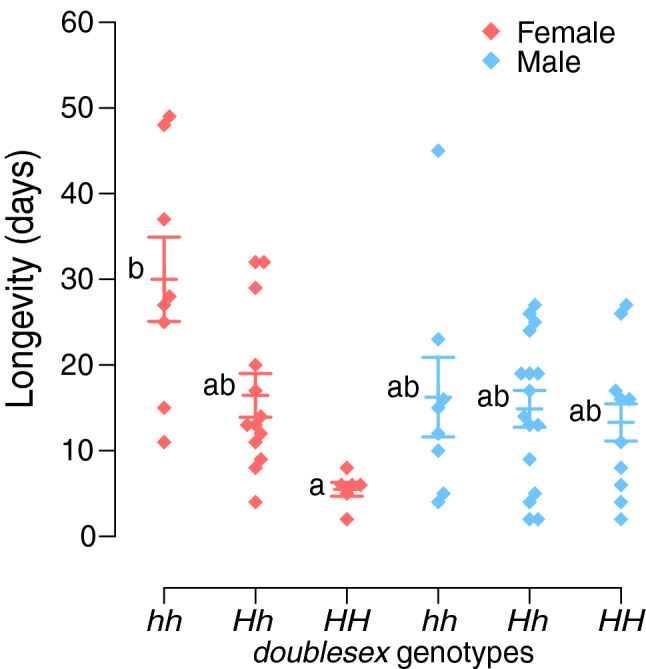
Adult longevity in each *doublesex* genotype of *Papilio polytes*. We used a cross between a wild-caught male adult (*dsx* genotype, *Hh*) and a wild-caught virgin female (*dsx* genotype, *Hh*) collected as a pupa. Red plots, females; blue plots, males. Middle bar represents mean, and upper and lower bars indicate standard error. Different letters indicate significant differences between groups (Tukey post hoc test, P < 0.05; Supplementary Table S6).

Finally, using a cross between females and males heterozygous for *dsx* (i.e. *Hh*), we compared the mortality, larval developmental duration, pupal period, adult forewing length, and adult lifespan amongst the three genotypes, namely *HH*, *Hh*, and *hh* (expected *HH*:*Hh*:*hh* ratio = 1:2:1), and between the two sexes (expected male:female ratio = 1:1). Larvae were reared on an artificial diet containing Insecta F-II (Nihonnosankogyo, Japan) and *Citrus natsudaidai* leaf powder under long-day conditions (light:dark = 16:8 h) at 25 °C. The mortality rate during the larval period was 39.6% (Table 1). The genotypic frequencies of dead and eclosed individuals were consistent with the expected 1:2:1 ratio (Table 1). The sex ratio of eclosed individuals was consistent with the expected 1:1 ratio, but more males than females eclosed as healthy individuals, excluding individuals with eclosion insufficiency, such as abnormal wings (68.6% males) (Table 1). Genotype and sex did not affect larval developmental duration, pupal period, and adult forewing length (Supplementary Fig. S2, Supplementary Tables S3, S4). Finally, we compared the adult lifespan amongst genotypes and sexes in healthy individuals and individuals with slight eclosion insufficiency (flight is possible, but the wings are not fully extended) (Table 1). We also used additional individuals from the laboratory populations (females, *hh*:*Hh* = 5:4; males, *hh*:*Hh* = 1:2) to increase the number of samples analysed. A total of 61 individuals (females, *hh*:*Hh*:*HH* = 8:13:6; males, *hh*:*Hh*:*HH* = 8:16:10) were used to measure adult lifespan. Differences in parental genotypes were included in the statistical model as a random effect. Parental genotypes, sexes, and their interaction explained differences in adult lifespan (Supplementary Tables S3, S4). Notably, adult lifespan of *HH* females was significantly shorter than that of *hh* females (Fig. 3; Tukey post hoc test, P = 0.003; Supplementary Table S6), but there was no difference in adult lifespan of males amongst the genotypes (Fig. 3, Supplementary Table S6). Adult lifespan of *hh* females was much longer than that of males, although this difference was not significant (Fig. 3, Supplementary Table S6). Of note, we estimated the adult lifespan using unmated females and males. However, almost all females are usually mated in the wild (e.g. in *Papilio memnon*^36^), then the longevity of mated females may also be explored for elucidating the effects of *dsx-H* on the adult lifespan. The effects of genotypes on adult lifespan were evident in females but not in males, which may be caused by differences in the expression patterns of *dsx* between sexes. However, the genotype of male parents negatively affected the number of eggs laid by females (Fig. 1, Supplementary Tables S1, S2, S5), suggesting defects in the male-specific isoform of *dsx-H*. Further analyses of expression patterns in other tissues related to adult longevity and reproduction are warranted to explain this sex-specific pattern.

**Table 1:** Numbers and *doublesex* genotypes of individuals reared and emerged as adults in the rearing experiment with *Papilio polytes*.

	*n*^a^	*HH*^a^	*Hh*^a^	*hh*^a^	*P*^b^
**Number of dead**					
1st instar	5	3	2	0	
2nd instar	0	0	0	0	
3rd instar	4	1	2	1	
4th instar	4	0	3	1	
5th (or 6th)^c^ instar	10 (4, 6)	3 (2, 1)	2 (1, 1)	5 (1, 4)	
Prepupa	8	3	3	2	
Pupa	7 (2, 5)	1 (0, 1)	4 (0, 4)	2 (2, 0)	
Total number of dead	38	11	16	11	0.62
**Number of emerged**					
Healthy individual	42 (28, 14)^d^	13 (9, 4)	19 (12, 7)	10 (7, 3)	
Slight eclosion insufficiency	7 (3, 4)	3 (1, 2)	4 (2, 2)	0	
Severe eclosion insufficiency	9 (3, 6)	1 (0, 1)	7 (3, 4)	1 (0, 1)	
Total number of emerged	58 (34, 24)^d^	17 (10, 7)	30 (17, 13)	11 (7, 4)	0.52
Total	96	28	46	22	0.63

^a^The numbers in parentheses are the number of males and females, respectively.

^b^*P*-values of the chi-square test for the goodness of fit with the expected segregation ratio (HH:Hh:hh = 1:2:1).

^c^Two individuals molt five times in the larval period abnormally.

^d^More male than female emerged in healthy individuals (binomial test; *P* = 0.0436), but not in total (binomial test; *P* = 0.237).

Furthermore, mimetic females, specifically the homozygous-dominant (*HH*) ones, showed reduced fitness due to low fertility and short longevity (Figs. 2, 3, Supplementary Tables S1–S6). Decreased adult egg production and lifespan in mimetic females may be due to defects in the pleiotropic functions of mimetic *dsx-H* involved in sex differentiation or other developmental processes. Two highly conserved domains (DNA-binding and oligomerisation domain) of Dsx are considered to play important roles in development^37^. In *Papilio polytes*, there are 14–15 amino acid changes (including one in the oligomerisation domain)^5^ between mimetic Dsx-H and non-mimetic Dsx-h, some of which may adversely affect important traits, such as reproductive traits. In addition, the regulatory regions for *dsx-H* and *dsx-h* differ considerably^4^; thus, a regulatory change in *dsx-H* may affect the expression control for normal development. Furthermore, genes downstream of Dsx, such as *Yolk protein* (*Yp*)*1–3* and *vitellogenin*, are associated with adult lifespan and/or fecundity in *Drosophila* and honeybees^38–41^. Moreover, a negative correlation between adult *Yp* expression and lifespan has been reported in both male and female *Drosophila melanogaster*^41^. This may be because *Yp* expression level determines fecundity in females, and lifespan is a trade-off for fecundity; however, how males without ovaries show a similar tendency remains unknown^42^. In *Papilio polytes*, decreased lifespan may not be a trade-off, because both egg production and lifespan of *HH* mimetic females were reduced. In honeybees, *vitellogenin* prolonged lifespan but relieved oxidative stress and suppressed immune function^38–40^. Further studies on the expression pattern of downstream genes of Dsx are warranted to elucidate the molecular mechanisms through which mimetic *dsx-H* produces physiological defects.

There were significant differences in larval survival rates amongst parental genotypes in crosses of all combinations of *dsx* genotypes (Fig. 2c, Supplementary Tables S1, S2, S5); however, similar to that in the closely related species *Papilio memnon*^42^, there were no differences in larval survival rates amongst genotypes in a cross between heterozygous females and males (i.e. *Hh*) (Table 1). As mentioned above, although parental genotypes likely play pivotal roles in offspring survival, we cannot conclude that offspring genotypes did not negatively affect larval survival, as insufficient sample size for a cross between heterozygous females and males might have biased our results. According to Katoh et al.^20^, *dsx-H* produces mild deleterious effects on larval survival; in that case, it may be difficult to detect this deleterious effect with insufficient sample size.

**Figure 4:**
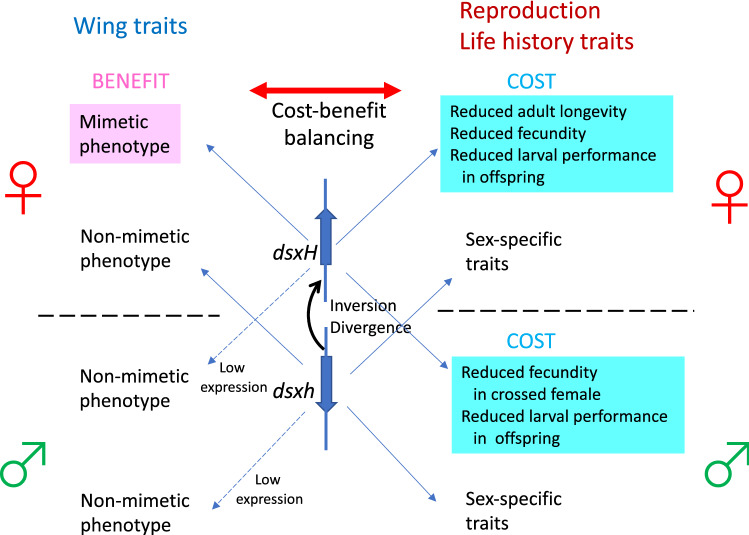
Model for *dsx-H* functions in the regulation of wing pattern in males and females as well as the reproductive and life history traits in males and females. Evolution of the mimetic allele of *dsx* is accompanied by physiological cost through pleiotropy. The cost–benefit balance affects the evolution and maintenance of mimicry.

In conclusion, Batesian mimicry in *Papilio polytes* has evolved via a chromosomal inversion and the divergence of the *dsx* locus. Simultaneously, the co-opted function of *dsx-H* for mimicry, which would affect the downstream gene network, may lead to physiological defects in mimetic females (specifically *HH* females) (Fig. 4). The benefit of mimicry is balanced with physiological costs, and the mimetic form is maintained when the benefits outweigh these costs. Therefore, such physiological costs may hinder the evolution of mimicry in *Papilio polytes* males, because males are subjected to lower predation pressure than females, which are less agile because of their larger abdomens^9^. However, our experiments show only the physiological costs in the laboratory, then in order to estimate the importance of physiological defects of *dsx-H* in the ecological contexts, comprehensive studies are needed to reveal the fitness differences among genotypes of *P*. *polytes* in the wild.

## Methods

### Study species and genotype frequencies in the wild

We purchased wild-caught *Papilio polytes* adults from Chokan-kabira (Okinawa, Japan) and identified *dsx* genotypes of mimetic females and males^4^, which were collected from Ishigaki Island, Okinawa, Japan, from April to September 2018 and from May 2017 to March 2018, respectively. To identify *dsx* genotypes, primers was designed based on the sequence of intron between exons 1 and 2 of *dsx*, as described elsewhere^4^. We designed two sets of primers by amplifying a region including an indel and determined the genotype based on the length of the PCR product. “Pp_dsx_hetero_primer_8_3” yielded products of 192 and 162 bp for the *H* and *h* alleles, respectively, and “Pp_dsx_hetero_primer_8_5” yielded products of 714 and 910 bp for the *H* and *h* alleles, respectively (Pp_dsx_hetero_primer_8_3, forward: 5′-AACTAGCGGTCGTAGGTTCG-3′, reverse: 5′-CATGCATTATTGAAAATTCCAG-3′; Pp_dsx_hetero_primer_8_5, forward: 5′-GCGCCCTTTCAATACCAGATTA-3′, reverse: 5′-GTGGTTGCCCACTGATCAAAAT-3′). PCR amplification was performed using KOD FX Neo (TOYOBO, Osaka) according to the manufacturer’s protocol with a 10 µL reaction system containing 9 μL PCR reaction mix (5 μL 2 × KOD FX Neo Buffer, 2 μL 2 mM dNTP mix, 0.15 μL 10 mM 5′-primer, 0.15 μL 10 mM 3′-primer, 0.2 μL 1 U KOD FX Neo DNA polymerase and 1.5 μL water) and 1 μL DNA template. PCR products were electrophoresed on 1%–3% agarose gels and stained with ethidium bromide. A single band was generated for homozygous individuals (*HH/hh*), and two bands were detected for heterozygous individuals (*Hh*). The frequencies of different *dsx* genotypes in males were evaluated based on the Hardy–Weinberg equilibrium using the R package HardyWeinberg^43,44^.

### Number of eggs laid, hatching rate, and larval survival rate

We used crosses between females and males with all combinations of *dsx* genotypes (*hh♂* × *hh♀*, *Hh♂* × *hh♀*, *HH♂* × *hh♀*, *hh♂* × *Hh♀*, *Hh♂* × *Hh♀*, *HH♂* × *Hh♀*, *hh♂* × *HH♀*, *Hh♂* × *HH♀*, and *HH♂* × *HH♀*) and examined the effects parental genotype on the number of eggs laid, hatching rate, and larval survival rate. First, wild females (purchased from Chokan-kabira) captured from Ishigaki Island were allowed to lay eggs in the laboratory, and the larvae that emerged from these eggs were reared under long-day conditions (light:dark = 16:8 h) at 25 °C to obtain the three *dsx* genotypes: *HH*, *Hh,* and *hh*. Parental female butterflies were captured in the wild, and the experimental populations were not inbred. Next, three males and three females were selected for each combination of *dsx* genotypes 2–3 days after eclosion and hand paired to mate (n = 27 pairs). To measure the number of eggs laid, we conducted egg collection trials. Mated females were individually placed in plastic cases (6 L) containing citrus leaves (*Citrus natsudaidai*) for 2 h at 25 °C under fluorescent light. After a trial, females were kept in an incubator at 15 °C for 2–3 days. Each female was fed a sports drink (Calpis, Asahi, Japan) before and after the trials. We repeated this trial until the death of the females and counted the number of eggs laid throughout the lifespan. Two to four trials were conducted for each female.

All laid eggs (n = 2286) were maintained under long-day conditions at 25 °C. Eggs that did not hatch within 7 days were assumed dead, and the hatching rate was calculated. After emergence, the larvae (n = 1767) were reared on an artificial diet (5.6 mL of water, 144 µL of a 10% formalin solution, 20 µg of chloramphenicol, 0.8 g for 1st to 2nd instar larvae and 1.2 g for 3rd to 5th instar larvae Insecta F-II (Nihonnosankogyo), and 0.8 g for 1st to 2nd instar larvae and 0.4 g for 3rd to 5th instar larvae *Citrus natsudaidai* leaf powder) under long-day conditions at 25 °C^4^. Finally, we calculated the survival rate until pupation (1017 individuals). Of note, our experiments were performed using an artificial diet. Therefore, there may be some biases in our results that are not observed in natural populations. For example, the survival rate in our experiments may be relatively lower than that in a natural environment. However, all our experiments were performed using the same artificial diet; therefore, there should be no significant problem in examining the effects of genotypes and/or sexes on various traits. These experiments and rearing were conducted from July to September 2016 and from June to August 2017.

### Larval development and adult lifespan

We used a cross between females and males that were heterozygous for the *dsx* allele (i.e. *Hh*) and reared the emerged larvae until death to explore the effects of genotypes and sex on larval survival, larval developmental duration, pupal period, adult forewing length, and adult lifespan. We purchased a wild-caught adult male (*Hh*) and a wild-caught pupa female (*Hh*) from Chokan-kabira, and performed forced mating by hand pairing to obtain larval populations of the three genotypes: *HH*, *Hh*, and *hh* (expected *HH*:*Hh*:*hh* ratio = 1:2:1). The mated female was placed in a plastic case (6 L) containing citrus leaves (*Citrus natsudaidai*) at 25 °C under fluorescent light, and a total of 97 eggs were obtained. The emerged larvae (n = 96) were reared on the artificial diet described above under long-day conditions at 25 °C and 40–50% humidity. First to fourth instar larvae were individually kept in small Petri dishes (diameter, 55 mm; height, 17 mm), and final-instar larvae were individually kept in large Petri dishes (diameter, 90 mm; height, 20 mm). The diet was changed once every 2 days. We measured pupal weight (to 0.1 mg) with an electric balance. Larval developmental duration was defined as the number of days from emergence to pupation, and pupal period was defined as the number of days from pupation to adult eclosion. For adults, we measured forewing length (to 0.1 mm) and divided them into three classes: healthy individuals, individuals with slight eclosion insufficiency (flight is possible, but the wings are not fully extended), and individuals with severe eclosion insufficiency (flight is impossible). To measure adult lifespan, healthy individuals and individuals with slight eclosion insufficiency were divided by sex in two cages (45 × 45 × 88 cm^3^) under long-day conditions (light:dark = 14:10 h) at 25 ± 1 °C and 30–40% humidity. Additional individuals from laboratory populations (females, *hh*:*Hh* = 5:4; males, *hh*:*Hh* = 1:2) were used to measure adult lifespan. Each female was fed a sports drink (Calpis). Mortality was checked twice a day (8:00–10:00 and 17:00–20:00), and the number of days until death was counted.

### Statistical analysis

In the experiment of crosses with all combinations of *dsx* genotypes, we explored the effects of parental genotypes on the number of eggs laid, hatching rate, and larval survival rate using a generalised linear model (GLM). The distributions assumed in GLM were selected based on the nature of response variables^45,46^. The number of eggs laid was analysed assuming a Poisson distribution, and the number of egg collection trials was incorporated into the model as an offset variable. The hatching and larval survival rates were analysed assuming a binomial distribution. The models with an optimal set of explanatory variables were identified using the “dredge” function in R (version 4.0.2)^43^ package MuMIn (version 1.43.17)^47^, which uses the lowest Akaike’s information criterion (AIC) to rank all possible models with all possible combinations of explanatory variables in the full model. Tukey post hoc tests were used to detect differences between groups using the “glht” function in R package multcomp^43,48^.

In a cross between females and males that were heterozygous for *dsx* (i.e. *Hh*), the observed ratios of the three *dsx* genotypes (*HH*:*Hh*:*hh*) were compared with the expected ratio of 1:2:1 under the null hypothesis assuming a random association of alleles and no mortality difference amongst the genotypes using the chi-square test for the goodness of fit in R^43^. We explored the effects of genotypes and sex on larval developmental duration, pupal period, and adult forewing length using a GLM. In the analyses of larval developmental duration and pupal period, we compared the AIC amongst the full models assuming three possible distributions (normal, gamma, and Poisson distributions) to select the optimal distribution. Then, we used gamma distribution in GLMs of larval developmental duration and pupal period. The AIC values of models for larval developmental period assuming normal, gamma, and Poisson distributions were 286.25, 281.74, and 290.66, respectively, and the AIC values of models for pupal period assuming normal, Gamma, and Poisson distributions were 101.22, 101.14, and 228.95, respectively. In the analysis of adult forewing length, we compared AIC between the full models assuming normal and gamma distributions and selected normal distribution. The AIC values of models for adult forewing length assuming normal and gamma distributions were 266.84 and 270.72, respectively. Model selection was performed as described above.

Adult lifespan was analysed using a generalised linear mixed model (GLMM). Differences in parental genotypes were included as a random effect. We compared the AIC amongst the full models assuming three possible distributions (normal, gamma, and Poisson distributions) to select the optimal distribution. Then, we used a normal distribution in the GLMM. The AIC values of models for adult lifespan assuming normal, gamma, and Poisson distributions were 435.27, 445.48, and 566.96, respectively. The GLMM analyses were performed using the R package lme4^43,49^. Model selection and Tukey post hoc tests were performed as described above.

## Supplementary information

Supplementary Information 1.

Supplementary Information 2.

Supplementary Information 3.

Supplementary Information 4.

## Data Availability

All data used in this study are available within the manuscript and its supplementary materials.
