# Supplementary material for: Batesian mimicry has evolved with deleterious effects of the pleiotropic gene doublesex
Source: Sci Rep. 2020 Dec 7;10:21333. doi: 10.1038/s41598-020-78055-1 (PMC7721872; doi:10.1038/s41598-020-78055-1)
Supplement: Supplementary file 1 — Supplementary Information 1. [file 41598_2020_78055_MOESM1_ESM.docx]

**Description of Additional Supplementary Files for:**

**Batesian mimicry has evolved with deleterious effects of the pleiotropic gene *doublesex***

Shinya Komata^1^, Tasuku Kitamura^1^ & Haruhiko Fujiwara^1^

^1^Department of Integrated Biosciences, Graduate School of Frontier Sciences, The University of Tokyo, Kashiwa, Chiba 277-8562, Japan.

File Name: Supplementary Data 1
Description: Row data of the number of eggs laid, hatching rate, and larval survival rate reported in Figure 2.

File Name: Supplementary Data 2
Description: Row data of forewing length, larval period, pupal period and adult lifespan reported in Figures S2 and 3.
