# Supplementary material for: Batesian mimicry has evolved with deleterious effects of the pleiotropic gene doublesex
Source: Sci Rep. 2020 Dec 7;10:21333. doi: 10.1038/s41598-020-78055-1 (PMC7721872; doi:10.1038/s41598-020-78055-1)
Supplement: Supplementary file 4 — Supplementary Information 4. [file 41598_2020_78055_MOESM4_ESM.docx]

**Supplementary Information for:**

**Batesian mimicry has evolved with deleterious effects of the pleiotropic gene *doublesex***

Shinya Komata^1^, Tasuku Kitamura^1^ & Haruhiko Fujiwara^1^

^1^Department of Integrated Biosciences, Graduate School of Frontier Sciences, The University of Tokyo, Kashiwa, Chiba 277-8562, Japan.

**Supplementary figure**

**Supplementary Figure S1. Illustrations of experimental design.** At first, we explored the *dsx* genotypic frequency of individuals collected in the wild (a). Next, we compared the number of eggs laid, hatching rate and survival rate in lava using crosses between females and males in all combinations of *dsx* genotypes (b). At last, using a cross between females and males that were heterozygous of *dsx* (i.e. genotype *Hh*) we compared mortality, larval development time, pupal weight, forewing length and adult life span among three genotypes: *HH*, *Hh*, *hh* and between the sexes (c).

**Supplementary Figure S2. Larval development time (a), pupal period (b) and adult forewing length (c) in each *doublesex* genotype of *Papilio poletes*.**

Red points, females; blue points, males. The middle bar represents the mean value, while the upper and lower bars denote standard errors.

**Supplementary Tables**

**Supplementary Table S1. Model selection of the generalised linear model (GLM) analyses incorporating the mean number of eggs laid, hatching rate and survival in *Papilio polytes*.**

| models | d.f. | LogLik | AIC | ∆AIC | weight |
| --- | --- | --- | --- | --- | --- |
| *Mean number of egg laid in each trial (GLM with a Poisson distribution)* | | | | | |
| Genotypes of female parent (F),  Genotypes of male parent (M), F × M | 9 | -105.255 | 228.510 | 0.000 | 0.998 |
| F, M | 5 | -115.722 | 241.443 | 12.933 | 0.002 |
| F | 3 | -137.984 | 281.968 | 53.458 | 0.000 |
| M | 3 | -144.252 | 294.504 | 65.994 | 0.000 |
| intercept-only model (null model) | 1 | -168.613 | 339.226 | 110.716 | 0.000 |
| *Hatching rate (GLM with a binomial distribution)* | | | | | |
| F, M, F × M | 9 | -75.311 | 168.623 | 0.000 | 0.874 |
| F, M | 5 | -81.305 | 172.611 | 3.988 | 0.119 |
| F | 3 | -86.151 | 178.303 | 9.680 | 0.007 |
| M | 3 | -97.345 | 200.691 | 32.068 | 0.000 |
| intercept-only model (null model) | 1 | -103.834 | 209.668 | 41.046 | 0.000 |
| *Survival rate (GLM with a binomial distribution)* | | | | | |
| F, M, F × M | 9 | -73.753 | 165.505 | 0.000 | 1.000 |
| F, M | 5 | -91.629 | 193.258 | 27.753 | 0.000 |
| M | 3 | -111.092 | 228.184 | 62.679 | 0.000 |
| F | 3 | -146.516 | 299.033 | 133.527 | 0.000 |
| intercept-only model (null model) | 1 | -170.733 | 343.466 | 177.961 | 0.000 |

d. f., degrees of freedom; LogLik, log-likelihood; AIC, Akaike’s information criterion; ∆AIC, difference in AIC of the best model. Models are ranked according to their Akaike weight (weight).

**Supplementary Table S2. Parameter estimates of the best model GLM selected by AIC (Table S1) on the mean number of eggs laid, hatching rate and survival in *Papilio polytes*.**

|  | Estimate | Error | *z* | *P* |
| --- | --- | --- | --- | --- |
| *Mean number of eggs laid* |  |  |  |  |
| Intercept | 3.838 | 0.042 | 90.572 | < 0.001 |
| Female parent [*Hh*] | -0.096 | 0.076 | -1.267 | 0.205 |
| Female parent [*HH*] | -0.653 | 0.093 | -7.001 | < 0.001 |
| Male parent [*Hh*] | -0.294 | 0.065 | -4.538 | < 0.001 |
| Male parent [*HH*] | -0.521 | 0.076 | -6.831 | < 0.001 |
| Female parent [*Hh*]*Male parent [*Hh*] | 0.064 | 0.115 | 0.560 | 0.575 |
| Female parent [*HH*]*Male parent [*Hh*] | 0.393 | 0.132 | 2.981 | 0.003 |
| Female parent [*Hh*]*Male parent [*HH*] | 0.019 | 0.128 | 0.145 | 0.885 |
| Female parent [*HH*]*Male parent [*HH*] | 0.582 | 0.139 | 4.196 | < 0.001 |
|  |  |  |  |  |
| *Hatching rate* |  |  |  |  |
| Intercept | 1.303 | 0.103 | 12.605 | < 0.001 |
| Female parent [*Hh*] | -0.068 | 0.183 | -0.373 | 0.709 |
| Female parent [*HH*] | 0.315 | 0.246 | 1.278 | 0.201 |
| Male parent [*Hh*] | 0.534 | 0.176 | 3.034 | 0.002 |
| Male parent [*HH*] | -0.268 | 0.178 | -1.509 | 0.131 |
| Female parent [*Hh*]*Male parent [*Hh*] | -0.555 | 0.286 | -1.939 | 0.053 |
| Female parent [*HH*]*Male parent [*Hh*] | -1.020 | 0.339 | -3.009 | 0.003 |
| Female parent [*Hh*]*Male parent [*HH*] | -0.361 | 0.288 | -1.254 | 0.210 |
| Female parent [*HH*]*Male parent [*HH*] | -0.846 | 0.330 | -2.560 | 0.010 |
|  |  |  |  |  |
| *Survival* |  |  |  |  |
| Intercept | 1.033 | 0.109 | 9.510 | < 0.001 |
| Female parent [*Hh*] | -0.849 | 0.180 | -4.716 | < 0.001 |
| Female parent [*HH*] | -1.932 | 0.228 | -8.472 | < 0.001 |
| Male parent [*Hh*] | -0.029 | 0.161 | -0.178 | 0.859 |
| Male parent [*HH*] | -1.352 | 0.185 | -7.311 | < 0.001 |
| Female parent [*Hh*]*Male parent [*Hh*] | 0.197 | 0.271 | 0.726 | 0.468 |
| Female parent [*HH*]*Male parent [*Hh*] | 0.259 | 0.321 | 0.807 | 0.419 |
| Female parent [*Hh*]*Male parent [*HH*] | 0.821 | 0.311 | 2.645 | 0.008 |
| Female parent [*HH*]*Male parent [*HH*] | 1.915 | 0.342 | 5.592 | < 0.001 |

**Supplementary Table S3. Model selection of generalized linear model (GLM) and generalized linear mixed model (GLMM) analyses on larval period, pupal period, forewing length and longevity in *Papilio polytes*.**

| models | d.f. | LogLik | AIC | ∆AIC | weight |
| --- | --- | --- | --- | --- | --- |
| *Larval period (GLM with a Gamma distribution)* | | | | | |
| intercept-only model (null model) | 2 | -136.005 | 276.011 | 0.000 | 0.464 |
| Sex | 3 | -135.359 | 276.718 | 0.707 | 0.326 |
| Genotype | 4 | -135.505 | 279.011 | 3.000 | 0.103 |
| Genotype, Sex | 5 | -134.752 | 279.504 | 3.494 | 0.081 |
| Genotype, Sex, Genotype × Sex | 7 | -133.870 | 281.740 | 5.729 | 0.026 |
| *Pupal period (GLM with a Gamma distribution)* | | | | | |
| intercept-only model (null model) | 2 | -44.047 | 92.095 | 0.000 | 0.605 |
| Sex | 3 | -43.988 | 93.975 | 1.880 | 0.236 |
| Genotype | 4 | -43.761 | 95.522 | 3.428 | 0.109 |
| Genotype, Sex | 5 | -43.680 | 97.360 | 5.265 | 0.043 |
| Genotype, Sex, Genotype × Sex | 7 | -43.571 | 101.141 | 9.047 | 0.007 |
| *Forewing length (GLM with a normal distribution)* | | | | | |
| Sex | 3 | -127.901 | 261.802 | 0.000 | 0.428 |
| intercept-only model (null model) | 2 | -128.934 | 261.867 | 0.065 | 0.414 |
| Genotype, Sex | 5 | -127.835 | 265.670 | 3.868 | 0.062 |
| Genotype | 4 | -128.853 | 265.705 | 3.903 | 0.061 |
| Genotype, Sex, Genotype × Sex | 7 | -126.420 | 266.839 | 5.037 | 0.035 |
| *Longevity (GLMM with a normal distribution. Parents was included as a random effect.)* | | | | | |
| Genotype, Sex, Genotype × Sex | 8 | -209.561 | 435.121 | 0.000 | 0.998 |
| Genotype, Sex | 6 | -218.174 | 448.348 | 13.227 | 0.001 |
| Genotype | 5 | -220.078 | 450.156 | 15.035 | 0.001 |
| Sex | 4 | -225.392 | 458.783 | 23.662 | 0.000 |
| intercept-only model (null model) | 3 | -227.323 | 460.645 | 25.524 | 0.000 |

d. f., degrees of freedom; LogLik, log-likelihood; AIC, Akaike’s information criterion; ∆AIC, difference in AIC of the best model. Models are ranked according to their Akaike weight (weight).

**Supplementary Table S4. Parameter estimates of the best model GLM and GLMM selected by AIC (Table S3) on forewing length and adult longevity in *Papilio polytes*.**

|  | Estimate | Error | *t* | *P* |
| --- | --- | --- | --- | --- |
| *GLM: Forewing length* |  |  |  |  |
| Intercept | 39.837 | 0.792 | 50.292 | < 0.001 |
| Sex [male] | -1.417 | 0.996 | -1.422 | 0.162 |
|  |  |  |  |  |
| *GLMM: Longevity* |  |  |  |  |
| Variance of the random effect (Parents) = 28.02; Std. Dev. = 5.29 | | | | |
| Intercept | 29.153 | 5.047 | 5.777 | 0.023 |
| Sex [male] | -10.364 | 5.015 | -2.067 | 0.043 |
| Genotype [*Hh*] | -11.390 | 4.391 | -2.594 | 0.012 |
| Genotype [*HH*] | -20.267 | 5.509 | -3.679 | 0.001 |
| Sex [male]*Genotype [*Hh*] | 10.015 | 6.019 | 1.664 | 0.102 |
| Sex [male]*Genotype [*HH*] | 18.164 | 7.018 | 2.588 | 0.012 |

**Supplementary Table S5. *Post-hoc* Tukey test for the mean number of eggs laid, hatching rate and survival across the genotypes of female and male parents in *Papilio polytes*.**

| Contrasts  (Female. Male. - F. M.) | Estimate | Std. Error | *z* | *P* | 95% CI | |
| --- | --- | --- | --- | --- | --- | --- |
|  |  |  |  |  | Lower | Upper |
| *Mean number of eggs laid* |  |  |  |  |  |  |
| Hh.hh - hh.hh | -0.096 | 0.076 | -1.267 | 0.938 | -0.330 | 0.138 |
| HH.hh - hh.hh | -0.653 | 0.093 | -7.001 | **< 0.001** | -0.940 | -0.365 |
| hh.Hh - hh.hh | -0.294 | 0.065 | -4.538 | **< 0.001** | -0.494 | -0.094 |
| Hh.Hh - hh.hh | -0.326 | 0.082 | -3.963 | **0.002** | -0.580 | -0.072 |
| HH.Hh - hh.hh | -0.554 | 0.090 | -6.179 | **< 0.001** | -0.831 | -0.277 |
| hh.HH - hh.hh | -0.521 | 0.076 | -6.831 | **< 0.001** | -0.757 | -0.286 |
| Hh.HH - hh.hh | -0.599 | 0.091 | -6.562 | **< 0.001** | -0.881 | -0.317 |
| HH.HH - hh.hh | -0.592 | 0.091 | -6.508 | **< 0.001** | -0.873 | -0.311 |
| HH.hh - Hh.hh | -0.557 | 0.104 | -5.344 | **< 0.001** | -0.878 | -0.235 |
| hh.Hh - Hh.hh | -0.198 | 0.080 | -2.486 | 0.230 | -0.444 | 0.048 |
| Hh.Hh - Hh.hh | -0.230 | 0.094 | -2.435 | 0.255 | -0.522 | 0.062 |
| HH.Hh - Hh.hh | -0.458 | 0.101 | -4.536 | **< 0.001** | -0.770 | -0.146 |
| hh.HH - Hh.hh | -0.425 | 0.089 | -4.761 | **< 0.001** | -0.701 | -0.150 |
| Hh.HH - Hh.hh | -0.503 | 0.102 | -4.911 | **< 0.001** | -0.819 | -0.187 |
| HH.HH - Hh.hh | -0.496 | 0.102 | -4.857 | **< 0.001** | -0.812 | -0.181 |
| hh.Hh - HH.hh | 0.358 | 0.096 | 3.715 | **0.006** | 0.061 | 0.656 |
| Hh.Hh - HH.hh | 0.327 | 0.109 | 2.997 | 0.064 | -0.010 | 0.663 |
| HH.Hh - HH.hh | 0.098 | 0.115 | 0.859 | 0.995 | -0.255 | 0.452 |
| hh.HH - HH.hh | 0.131 | 0.105 | 1.255 | 0.941 | -0.191 | 0.454 |
| Hh.HH - HH.hh | 0.054 | 0.116 | 0.463 | 1.000 | -0.304 | 0.411 |
| HH.HH - HH.hh | 0.060 | 0.116 | 0.520 | 1.000 | -0.297 | 0.417 |
| Hh.Hh - hh.Hh | -0.032 | 0.086 | -0.370 | 1.000 | -0.297 | 0.233 |
| HH.Hh - hh.Hh | -0.260 | 0.093 | -2.794 | 0.111 | -0.547 | 0.027 |
| hh.HH - hh.Hh | -0.227 | 0.080 | -2.830 | 0.102 | -0.475 | 0.021 |
| Hh.HH - hh.Hh | -0.305 | 0.095 | -3.222 | **0.033** | -0.597 | -0.013 |
| HH.HH - hh.Hh | -0.298 | 0.094 | -3.160 | **0.040** | -0.589 | -0.007 |
| HH.Hh - Hh.Hh | -0.228 | 0.106 | -2.153 | 0.426 | -0.555 | 0.099 |
| hh.HH - Hh.Hh | -0.195 | 0.095 | -2.058 | 0.491 | -0.488 | 0.098 |
| Hh.HH - Hh.Hh | -0.273 | 0.107 | -2.543 | 0.203 | -0.604 | 0.058 |
| HH.HH - Hh.Hh | -0.266 | 0.107 | -2.487 | 0.230 | -0.597 | 0.064 |
| hh.HH - HH.Hh | 0.033 | 0.101 | 0.323 | 1.000 | -0.280 | 0.346 |
| Hh.HH - HH.Hh | -0.045 | 0.113 | -0.396 | 1.000 | -0.394 | 0.304 |
| HH.HH - HH.Hh | -0.038 | 0.113 | -0.339 | 1.000 | -0.387 | 0.310 |
| Hh.HH - hh.HH | -0.078 | 0.103 | -0.754 | 0.998 | -0.395 | 0.240 |
| HH.HH - hh.HH | -0.071 | 0.103 | -0.692 | 0.999 | -0.388 | 0.246 |
| HH.HH - Hh.HH | 0.007 | 0.114 | 0.057 | 1.000 | -0.346 | 0.359 |
| *Hatching rate* |  |  |  |  |  |  |
| Hh.hh - hh.hh | -0.068 | 0.183 | -0.373 | 1.000 | -0.632 | 0.496 |
| HH.hh - hh.hh | 0.315 | 0.246 | 1.278 | 0.935 | -0.446 | 1.075 |
| hh.Hh - hh.hh | 0.534 | 0.176 | 3.034 | 0.059 | -0.010 | 1.078 |
| Hh.Hh - hh.hh | -0.088 | 0.197 | -0.448 | 1.000 | -0.697 | 0.521 |
| HH.Hh - hh.hh | -0.171 | 0.211 | -0.809 | 0.996 | -0.823 | 0.481 |
| hh.HH - hh.hh | -0.268 | 0.178 | -1.509 | 0.846 | -0.817 | 0.281 |
| Hh.HH - hh.hh | -0.697 | 0.198 | -3.515 | **0.012** | -1.309 | -0.085 |
| HH.HH - hh.hh | -0.799 | 0.196 | -4.081 | **0.001** | -1.404 | -0.194 |
| HH.hh - Hh.hh | 0.383 | 0.269 | 1.420 | 0.886 | -0.449 | 1.215 |
| hh.Hh - Hh.hh | 0.602 | 0.207 | 2.906 | 0.084 | -0.038 | 1.243 |
| Hh.Hh - Hh.hh | -0.020 | 0.225 | -0.090 | 1.000 | -0.717 | 0.676 |
| HH.Hh - Hh.hh | -0.103 | 0.238 | -0.432 | 1.000 | -0.837 | 0.632 |
| hh.HH - Hh.hh | -0.200 | 0.209 | -0.959 | 0.989 | -0.844 | 0.444 |
| Hh.HH - Hh.hh | -0.629 | 0.226 | -2.778 | 0.118 | -1.328 | 0.070 |
| HH.HH - Hh.hh | -0.731 | 0.224 | -3.260 | **0.030** | -1.424 | -0.038 |
| hh.Hh - HH.hh | 0.220 | 0.265 | 0.829 | 0.996 | -0.599 | 1.039 |
| Hh.Hh - HH.hh | -0.403 | 0.280 | -1.442 | 0.877 | -1.266 | 0.460 |
| HH.Hh - HH.hh | -0.486 | 0.290 | -1.677 | 0.754 | -1.380 | 0.409 |
| hh.HH - HH.hh | -0.583 | 0.266 | -2.190 | 0.403 | -1.404 | 0.239 |
| Hh.HH - HH.hh | -1.012 | 0.280 | -3.609 | **0.009** | -1.877 | -0.146 |
| HH.HH - HH.hh | -1.114 | 0.279 | -3.999 | **0.002** | -1.974 | -0.253 |
| Hh.Hh - hh.Hh | -0.623 | 0.220 | -2.827 | 0.104 | -1.303 | 0.058 |
| HH.Hh - hh.Hh | -0.705 | 0.233 | -3.028 | 0.060 | -1.425 | 0.014 |
| hh.HH - hh.Hh | -0.802 | 0.203 | -3.954 | **0.002** | -1.429 | -0.176 |
| Hh.HH - hh.Hh | -1.231 | 0.221 | -5.565 | **< 0.001** | -1.915 | -0.548 |
| HH.HH - hh.Hh | -1.334 | 0.219 | -6.087 | **< 0.001** | -2.010 | -0.657 |
| HH.Hh - Hh.Hh | -0.083 | 0.249 | -0.331 | 1.000 | -0.852 | 0.687 |
| hh.HH - Hh.Hh | -0.180 | 0.221 | -0.811 | 0.996 | -0.864 | 0.504 |
| Hh.HH - Hh.Hh | -0.609 | 0.238 | -2.554 | 0.200 | -1.345 | 0.128 |
| HH.HH - Hh.Hh | -0.711 | 0.236 | -3.008 | 0.063 | -1.441 | 0.019 |
| hh.HH - HH.Hh | -0.097 | 0.234 | -0.415 | 1.000 | -0.820 | 0.626 |
| Hh.HH - HH.Hh | -0.526 | 0.250 | -2.104 | 0.461 | -1.298 | 0.246 |
| HH.HH - HH.Hh | -0.628 | 0.248 | -2.532 | 0.210 | -1.395 | 0.138 |
| Hh.HH - hh.HH | -0.429 | 0.222 | -1.929 | 0.584 | -1.116 | 0.258 |
| HH.HH - hh.HH | -0.531 | 0.220 | -2.412 | 0.270 | -1.211 | 0.149 |
| HH.HH - Hh.HH | -0.102 | 0.237 | -0.431 | 1.000 | -0.835 | 0.631 |
| *Survival* |  |  |  |  |  |  |
| Hh.hh - hh.hh | -0.849 | 0.180 | -4.716 | **< 0.001** | -1.404 | -0.293 |
| HH.hh - hh.hh | -1.932 | 0.228 | -8.472 | **< 0.001** | -2.636 | -1.228 |
| hh.Hh - hh.hh | -0.029 | 0.161 | -0.178 | 1.000 | -0.527 | 0.469 |
| Hh.Hh - hh.hh | -0.681 | 0.196 | -3.474 | **0.014** | -1.286 | -0.076 |
| HH.Hh - hh.hh | -1.701 | 0.221 | -7.710 | **< 0.001** | -2.383 | -1.020 |
| hh.HH - hh.hh | -1.352 | 0.185 | -7.311 | **< 0.001** | -1.924 | -0.781 |
| Hh.HH - hh.hh | -1.380 | 0.231 | -5.969 | **< 0.001** | -2.093 | -0.666 |
| HH.HH - hh.hh | -1.369 | 0.234 | -5.857 | **< 0.001** | -2.091 | -0.647 |
| HH.hh - Hh.hh | -1.083 | 0.247 | -4.394 | **< 0.001** | -1.845 | -0.322 |
| hh.Hh - Hh.hh | 0.820 | 0.187 | 4.394 | **< 0.001** | 0.244 | 1.396 |
| Hh.Hh - Hh.hh | 0.168 | 0.217 | 0.772 | 0.997 | -0.503 | 0.839 |
| HH.Hh - Hh.hh | -0.853 | 0.240 | -3.557 | **0.011** | -1.593 | -0.112 |
| hh.HH - Hh.hh | -0.504 | 0.207 | -2.430 | 0.258 | -1.144 | 0.137 |
| Hh.HH - Hh.hh | -0.531 | 0.249 | -2.129 | 0.441 | -1.301 | 0.239 |
| HH.HH - Hh.hh | -0.521 | 0.252 | -2.067 | 0.485 | -1.298 | 0.257 |
| hh.Hh - HH.hh | 1.903 | 0.233 | 8.157 | **< 0.001** | 1.183 | 2.623 |
| Hh.Hh - HH.hh | 1.251 | 0.258 | 4.840 | **< 0.001** | 0.453 | 2.049 |
| HH.Hh - HH.hh | 0.231 | 0.278 | 0.830 | 0.996 | -0.627 | 1.088 |
| hh.HH - HH.hh | 0.579 | 0.250 | 2.315 | 0.322 | -0.193 | 1.352 |
| Hh.HH - HH.hh | 0.552 | 0.286 | 1.930 | 0.581 | -0.331 | 1.436 |
| HH.HH - HH.hh | 0.563 | 0.288 | 1.952 | 0.565 | -0.327 | 1.453 |
| Hh.Hh - hh.Hh | -0.652 | 0.202 | -3.226 | **0.033** | -1.276 | -0.028 |
| HH.Hh - hh.Hh | -1.672 | 0.226 | -7.397 | **< 0.001** | -2.371 | -0.974 |
| hh.HH - hh.Hh | -1.324 | 0.191 | -6.914 | **< 0.001** | -1.915 | -0.732 |
| Hh.HH - hh.Hh | -1.351 | 0.236 | -5.715 | **< 0.001** | -2.081 | -0.621 |
| HH.HH - hh.Hh | -1.340 | 0.239 | -5.610 | **< 0.001** | -2.078 | -0.603 |
| HH.Hh - Hh.Hh | -1.020 | 0.252 | -4.049 | **0.001** | -1.799 | -0.242 |
| hh.HH - Hh.Hh | -0.672 | 0.221 | -3.033 | 0.058 | -1.355 | 0.012 |
| Hh.HH - Hh.Hh | -0.699 | 0.261 | -2.675 | 0.150 | -1.506 | 0.108 |
| HH.HH - Hh.Hh | -0.688 | 0.264 | -2.612 | 0.174 | -1.502 | 0.126 |
| hh.HH - HH.Hh | 0.349 | 0.244 | 1.432 | 0.880 | -0.403 | 1.101 |
| Hh.HH - HH.Hh | 0.322 | 0.280 | 1.148 | 0.965 | -0.544 | 1.187 |
| HH.HH - HH.Hh | 0.332 | 0.282 | 1.176 | 0.959 | -0.540 | 1.204 |
| Hh.HH - hh.HH | -0.027 | 0.253 | -0.108 | 1.000 | -0.809 | 0.754 |
| HH.HH - hh.HH | -0.017 | 0.255 | -0.066 | 1.000 | -0.806 | 0.772 |
| HH.HH - Hh.HH | 0.010 | 0.291 | 0.036 | 1.000 | -0.887 | 0.908 |

**Supplementary Table S6. *Post-hoc* Tukey test for adult longevity across genotypes in *Papilio polytes*.**

| Contrasts (Male genotype – female genotype) | Estimate | Std. Error | *z* | *P* | 95% CI | |
| --- | --- | --- | --- | --- | --- | --- |
|  |  |  |  |  | Lower | Upper |
| m.hh - f.hh | -10.263 | 5.113 | -2.007 | 0.332 | -24.792 | 4.266 |
| f.Hh - f.hh | -11.121 | 4.591 | -2.423 | 0.145 | -24.167 | 1.925 |
| m.Hh - f.hh | -11.530 | 4.536 | -2.542 | 0.109 | -24.419 | 1.360 |
| f.HH - f.hh | -20.231 | 5.505 | -3.675 | **0.003** | -35.876 | -4.586 |
| m.HH - f.hh | -12.431 | 4.957 | -2.508 | 0.119 | -26.517 | 1.655 |
| f.Hh - m.hh | -0.858 | 4.310 | -0.199 | 1.000 | -13.107 | 11.391 |
| m.Hh - m.hh | -1.267 | 4.087 | -0.310 | 1.000 | -12.880 | 10.347 |
| f.HH - m.hh | -9.968 | 5.057 | -1.971 | 0.353 | -24.338 | 4.402 |
| m.HH - m.hh | -2.168 | 4.453 | -0.487 | 0.997 | -14.823 | 10.487 |
| m.Hh - f.Hh | -0.409 | 3.607 | -0.113 | 1.000 | -10.660 | 9.842 |
| f.HH - f.Hh | -9.110 | 4.708 | -1.935 | 0.375 | -22.490 | 4.270 |
| m.HH - f.Hh | -1.310 | 4.053 | -0.323 | 1.000 | -12.829 | 10.209 |
| f.HH - m.Hh | -8.701 | 4.472 | -1.946 | 0.369 | -21.411 | 4.009 |
| m.HH - m.Hh | -0.901 | 3.777 | -0.239 | 1.000 | -11.634 | 9.832 |
| m.HH - f.HH | 7.800 | 4.791 | 1.628 | 0.574 | -5.815 | 21.415 |
